# Novel Loss-of-Function Variant in HNF1a Induces β-Cell Dysfunction through Endoplasmic Reticulum Stress

**DOI:** 10.3390/ijms232113022

**Published:** 2022-10-27

**Authors:** Yinling Chen, Jianxin Jia, Qing Zhao, Yuxian Zhang, Bingkun Huang, Likun Wang, Juanjuan Tian, Caoxin Huang, Mingyu Li, Xuejun Li

**Affiliations:** 1 Department of Endocrinology and Diabetes, The First Affiliated Hospital of Xiamen University, School of Medicine, Xiamen University, Xiamen 361005, China; 2 Fujian Provincial Key Laboratory of Innovative Drug Target Research, School of Pharmaceutical Sciences and School of Life Sciences, Xiamen University, Xiamen 361102, China; 3 Fujian Province Key Laboratory of Diabetes Translational Medicine, Xiamen Diabetes Institute, Xiamen 361003, China

**Keywords:** HNF1a, β cell, insulin secretion, ER stress, variant

## Abstract

Heterozygous variants in the hepatocyte nuclear factor 1a (HNF1a) cause MODY3 (maturity-onset diabetes of the young, type 3). In this study, we found a case of novel HNF1a p.Gln125* (HNF1a-Q125ter) variant clinically. However, the molecular mechanism linking the new HNF1a variant to impaired islet β-cell function remains unclear. Firstly, a similar HNF1a-Q125ter variant in zebrafish (*hnf1a^+/−^*) was generated by CRISPR/Cas9. We further crossed *hnf1a^+/−^* with several zebrafish reporter lines to investigate pancreatic β-cell function. Next, we introduced HNF1a-Q125ter and HNF1a shRNA plasmids into the Ins-1 cell line and elucidated the molecular mechanism. *hnf1a^+/−^* zebrafish significantly decreased the β-cell number, insulin expression, and secretion. Moreover, β cells in *hnf1a^+/−^* dilated ER lumen and increased the levels of ER stress markers. Similar ER-stress phenomena were observed in an HNF1a-Q125ter-transfected Ins-1 cell. Follow-up investigations demonstrated that HNF1a-Q125ter induced ER stress through activating the PERK/eIF2a/ATF4 signaling pathway. Our study found a novel loss-of-function HNF1a-Q125ter variant which induced β-cell dysfunction by activating ER stress via the PERK/eIF2a/ATF4 signaling pathway.

## 1. Introduction

MODY3 (maturity-onset diabetes of the young, type 3) is the most common form of MODY, which is caused by heterozygous variants in hepatocyte nuclear factor 1 alpha (HNF1a) [1]. So far, more than 1200 various pathogenic and non-pathogenic HNF1a variants have been identified [2]. HNF1a plays a key role in the regulation of β-cell function, which not only controls cell lineage differentiation but also maintains β-cell identity [3,4]. Importantly, HNF1a is a master regulatory transcription factor that controls the expression of more than 106 target genes in human pancreatic islets [5], including SLC2A2 [6], PDX1 [7], and FOXA3 [8].

The histological analysis of one donor bearing a disease-causing HNF1aT260M variant showed normal β-cell and α-cell mass but impaired glucose-stimulated insulin secretion (GSIS) [9]. However, HNF1a knockout mice (*Hnf1a^−/−^*) displayed reduced β-cell mass and impaired GSIS, together with a low level of insulin [10,11,12,13]. Meanwhile, human iPS-derived β-like cells with a HNF1a^+/H126D^ variant had impaired GSIS after 6 months of transplantation into mice [14]. In line with those results, the dominant-negative HNF1a variants (P291fsinsC) impaired glucose-simulated insulin secretion [15].

Importantly, the endoplasmic reticulum (ER) plays a key role in insulin secretion because it controls insulin synthesis, proper folding, and response to glucose [16,17]. ER stress is caused by the disturbance of ER homeostasis, such as a sudden increase in proinsulin synthesis or disruption of ER Ca^2+^ homeostasis, which leads to the accumulation of unfolded or misfolded proteins in the ER lumen and activation of intracellular signaling pathways [18,19]. This response is collectively referred to as unfolded protein response (UPR) [20,21]. Indeed, most of the gene loci associated with monogenic diabetes are directly involved in the maintenance of β-cell function, and some of these genes are associated with ER-stress-mediated β-cell dysfunction [22,23,24].

Although it is well-known that many HNF1a variants lead to islet β-cell dysfunction and associate with MODY3, the detailed molecular mechanism still needs to be further illustrated. In this study, we found a novel heterozygous HNF1a variant in a human, HNF1a p.Gln125* (HNF1a-Q125ter). Then we generated a similar heterozygous HNF1a-Q125ter variant in zebrafish by CRISPR/Cas9 (*hnf1a^+/−^*). We also performed knockdown HNF1a and overexpression of HNF1a-Q125ter in Ins-1 cell lines. By using both in vivo and in vitro models, we illustrated the mechanism of HNF1a-Q125ter-induced β-cell dysfunction. Our results revealed that HNF1a-Q125ter impaired β-cell function by inducing ER stress through the PERK/eIF2a/ATF4 signaling pathway.

## 2. Results

### 2.1. Clinical and Structural Characterization of HNF1a-Q125ter Variant

**Figure 1:**
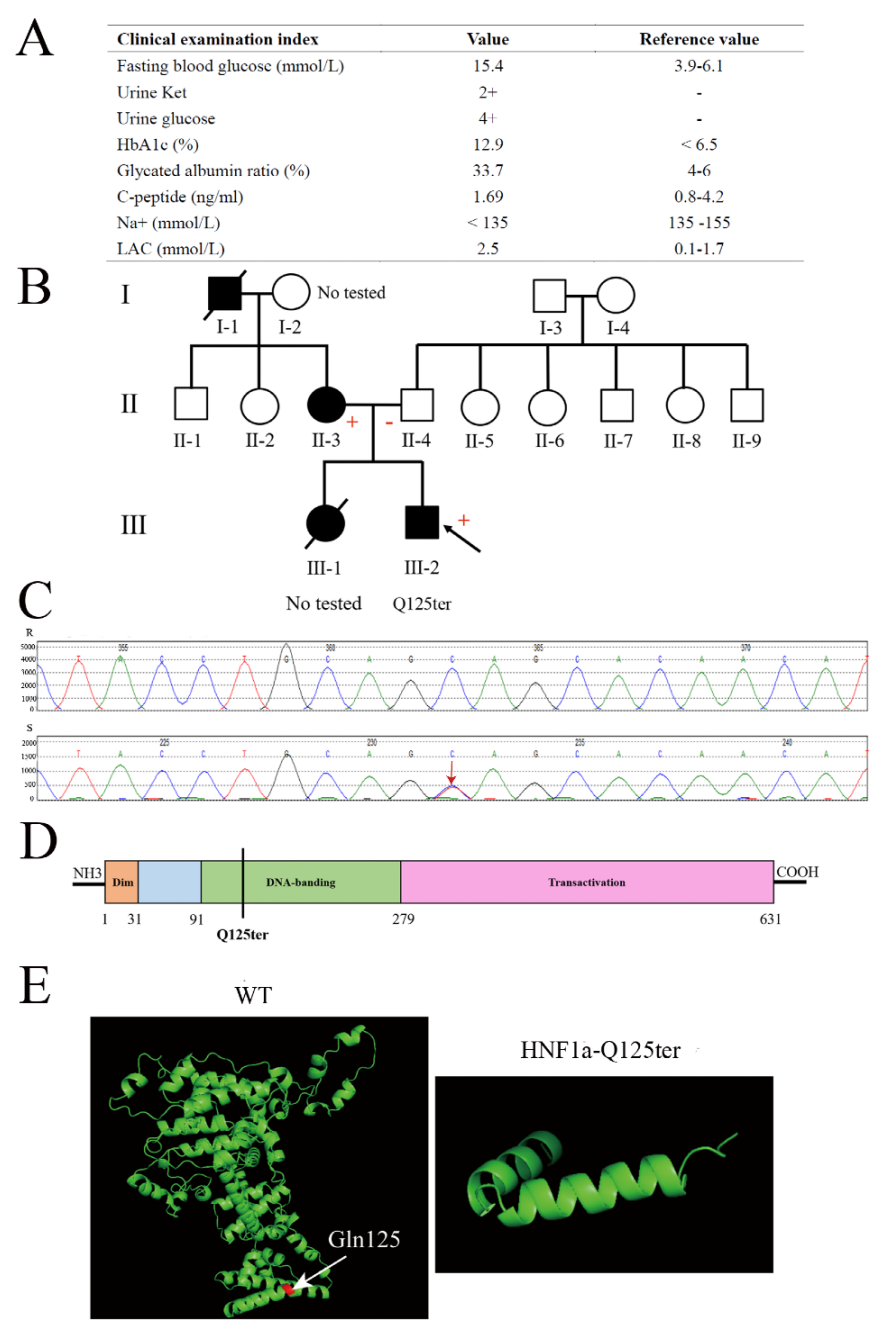
Clinical and functional characteristics of the HNF1a-Q125ter variant. (**A**) Clinical and biochemical indices of subject carrying the HNF1a-Q125ter variant. Urine glucose and urine ketosis levels suggested the patient had diabetic ketosis. Meanwhile, a high level of LAC indicated lactic acid metabolism disorder: +, positive; −, negative. (**B**) Pedigree. Circle, female; square, male; black slash segment, death; black arrow, proband. Those affected with diabetes are shaded black. Corresponding variant status, if known. Carriers of the mutation are marked with a (+). (**C**) Sanger sequencing map of HNF1a-Q125ter variant. Black arrow indicates heterozygous mutation for HNF1a: Exon2, c.373C>T, p.(Gln125*). (**D**) A diagram showing the HNF1a protein and domains. (**E**) Protein structure modeling of WT and HNF1a-Q125ter. White arrow indicates Gln125. HbA1c, hemoglobin A1c; LAC, lactic acid; Ket, ketosis.

A 15-year-old male adolescent was diagnosed with diabetes. He had a higher fasting blood glucose and HbA1c (15.4 mmol/L and 12.9%, respectively) (Figure 1A). Meanwhile, his urine glucose was 4+ and urine ketone was 2+, which suggested the patient might have diabetic ketosis. The normal reference for both is negative (−) (Figure 1A). Furthermore, the patient had a normal level of C-peptide (1.69 ng/mL), but a high level of lactic acid (LAC) (Figure 1A). As shown in Figure 1B, the patient’s mother had a family history of diabetes. According to these clinical genotypes, we speculated that the patient could have a MODY. Therefore, we used Sanger sequencing to identify the type of MODY in order to better treat the patient. The exon coding regions of ABCC8, AKT2, BLK, CEL, EIF2AK3, GCK, GLIS3, GLUD1, HADH, HNF1A, HNF1B, HNF4A, INS, INSR, KCNJ11, KLF11, MAPK8IP1, NEUROD1, PAX4, PDX1, PLAG1, PTF1A, RFX6, SLC19A2, SLC2A2, UCP2, and ZFP5 were directly sequenced. The result of Sanger sequencing showed that the 125th glutamine of the patient’s HNF1a had mutated into a stop codon (HNF1a p.Gln125*/HNF1a-Q125ter), which was a heterozygous variant (Figure 1B,C). As a consequence, the patient was diagnosed with MODY3. Meanwhile, the mother of the patient also had a HNF1a-Q125ter variant (Figure 1B). Interestingly, the HNF1a-Q125ter is a new variant that has never been reported. The HNF1a variant gene structure, functional domains, and location are represented in Figure 1D, which indicates that HNF1a-Q125ter could impair DNA-banding and transactivation domains of HNF1a. Additionally, HNF1a-Q125ter had an impaired protein structure, the specific results of which are shown in Figure 1E. Surprisingly, the patient did not respond to glimepiride, which is sulfonylureas.

### 2.2. Similar HNF1a-Q125ter Variant Impaired Pancreatic β-Cell Function in Zebrafish

**Figure 2:**
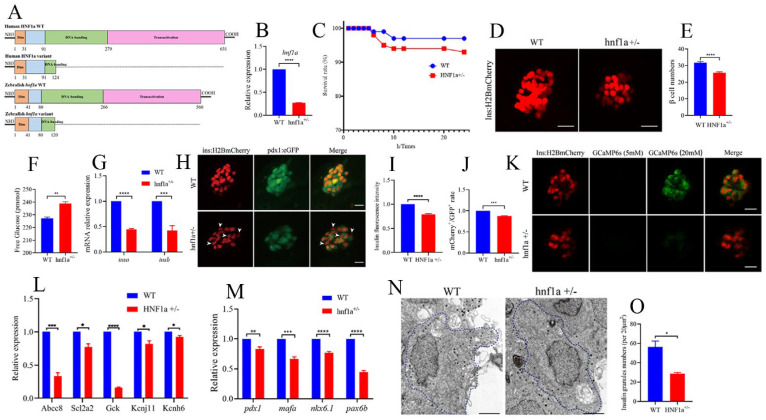
Similar variant impaired β-cell function in zebrafish. (**A**) Schematic representation of a similar HNF1a-Q125ter variant in zebrafish generated by CRISPR/Cas9. Mutation was caused via a two-base-pair deletion at the CRISPR target site for HNF1a. (**B**) The transcription level of hnf1a in WT and *hnf1a^+/−^*; *n* = 50 larvae for each genotype. (**C**) Survival curves of WT and *hnf1a^+/−^* zebrafish from 0 h to 24 h. (**D**,**E**) Representative confocal images (**D**) and quantification (**E**) of the β-cell number from Tg(−1.2ins:H2BmCherry) and *hnf1a^+/−^;* Tg(−1.2ins:H2BmCherry) zebrafish larvae. Scale bar indicates 25 μm; *n* = 20–35 larvae for each genotype. (**F**) Total free glucose level of WT and *hnf1a^+/−^* larvae at 6 dpf. (**G**) qRT-PCR analysis of the expression of *insa* and *insb* mRNA levels in WT and *hnf1a^+/−^* larvae at 6 dpf. (**H**) Representative confocal images of Tg(ins:H2BmCherry); Tg(pdx1:GFP) at 6 dpf for WT and *hnf1a^+/−^*. β-cells expressed insulin (mCherry^+^) without pdx1 (GFP^−^) are shown by white arrows. Scale bar: 10 μm. (**I**) Quantification of fluorescence intensity for insulin in WT and *hnf1a^+/−^*. (**J**) Quantification of double-positive cells’ (mCherry^+^/GFP^+^) rate in WT and *hnf1a^+/−^* larvae at 6 dpf; *n* = 3 larvae for each genotype. (**K**) Representative images of GCaMP6s response in β cells of Tg(Ins:H2BmCherry); Tg(Ins:GCaMP6s) and *hnf1a^−/−^*; Tg(Ins:H2BmCherry); Tg(Ins:GCaMP6s) by 5 or 20 mM glucose ECS solution; the green signal is GCaMP6s. Scale bar: 10 μm. (**L**) RT-qPCR quantification of mRNA levels for insulin-secretion markers in WT and *hnf1a^+/−^* larvae at 6 dpf: abcc8, scl2a2, gck, kcnj11, and kcnh6. (**M**) RT-qPCR quantification of mRNA levels for β-cell maturation and differentiation markers in WT and *hnf1a^+/−^* larvae at 6 dpf: mafa, pdx1, nkx6.1, and pax6b. (**N**,**O**) Representative electron micrographs of β cells (**N**) and quantification of insulin granules (**O**) in β cells from WT and *hnf1a^+/−^* larvae at 6 dpf. (**N**,**O**) Transmission electron microscopy for insulin granules. Islet isolation from WT and *hnf1a^+/−^* larvae at 6 dpf. The dotted blue lines represent intact individual β cells. Bar scale: 2 μm. *n* = 3 intact individual β cells for each genotype. Results are represented as means with standard errors; * *p* < 0.05, ** *p* < 0.01, *** *p* < 0.001, and **** *p* < 0.0001. Student’s *t*-test. All experiments were performed at least three times, unless otherwise indicated. WT, wild type.

In order to investigate the function of the HNF1a-Q125ter variant, we generated a zebrafish line with HNF1a containing a mutation at a similar position using CRISPR/Cas9. Details of the mutation position of HNF1a in zebrafish are represented in Figure 2A and Supplementary Figure S1A. The mRNA level of hnf1a decreased in *hnf1a^+/−^* (Figure 2B). Furthermore, the survival rate of *hnf1a^+/−^* larvae was lower than WT (Figure 2C). We also evaluated the morphological changes in *hnf1a^+/−^* zebrafish during the different development stages. However, there were no obvious morphological phenotype differences between WT and *hnf1a^+/−^* zebrafish at the stages of 1, 2, 3, 4, 5, and 6 dpf (Figure S1B).

Since MODY3 is caused by the dysfunction of pancreatic β cells, we then surveyed the β-cell function in *hnf1a^+/−^* zebrafish larvae. Interestingly, the β-cell number was significantly decreased in *hnf1a^+/−^* (Figure 2D,E), and its total free glucose level was significantly increased (Figure 2F). Moreover, the insulin mRNA levels (both *insa* and *insb*) were downregulated, as measured by RT-qPCR (Figure 2G), and the insulin protein level was decreased, as indicated by insulin immunostaining (Figure 2H,I). In addition, the mRNA levels of several markers of β-cell identity, such as *mafa, pdx1, nkx6.1*, and *pax6b*, were downregulated in the isolated *hnf1a^+/−^* zebrafish islets (Figure 2M). There were fewer double-positive β cells (mCherry^+^;eGFP^+^) in *hnf1a^+/−^*; Tg(-1.2ins:H2BmCherry); Tg(pdx1:eGFP) than in the WT background reporter, Tg(-1.2ins:H2BmCherry); Tg(pdx1:eGFP) (Figure 2H,J). However, the exocrine pancreas was not abnormal (Supplementary Figure S4). Consequently, these data suggested that *hnf1a^+/−^* zebrafish had impaired insulin synthesis. 

We also investigated insulin-secretion ability in the *hnf1a^+/−^* zebrafish. The transcription level of marker genes for insulin secretion, e.g., abcc8, scl2a2, gck, kcnj11, and kcnh6, were significantly decreased in the isolated *hnf1a^+/−^* zebrafish islets (Figure 2L). Next, to examine whether *hnf1a^+/−^* affects insulin secretion in real time, we performed live-imaging by using the calcium influx reporter line Tg(Ins:GCaMP6s), which was GCaMP6 calcium indicators combined with an insulin promoter. Finally, according to the change of cytosolic calcium concentration, it was converted into fluorescence signal. β-cells were labeled with a red nuclear marker, while the GCaMP6s fluorescence was present in the green channel. Remarkably, β cells of *hnf1a^+/−^* islets displayed a severely blunted calcium influx in response to glucose (Figure 2K and Supplementary Videos). We also performed transmission electron microscopy (TEM) to access the granule population in the *hnf1a^+/−^* zebrafish islets. As shown in Figure 2N,O, the number of insulin granules was lower than WT. Taken together, these data indicated that mutation of the HNF1a at a similar position to the HNF1a-Q125ter variant in zebrafish resulted in reduced β-cell numbers, suppressed insulin synthesis, and impaired insulin secretion.

### 2.3. Similar HNF1a-Q125ter Variant Induced β-Cell ER Stress in Zebrafish 

**Figure 3:**
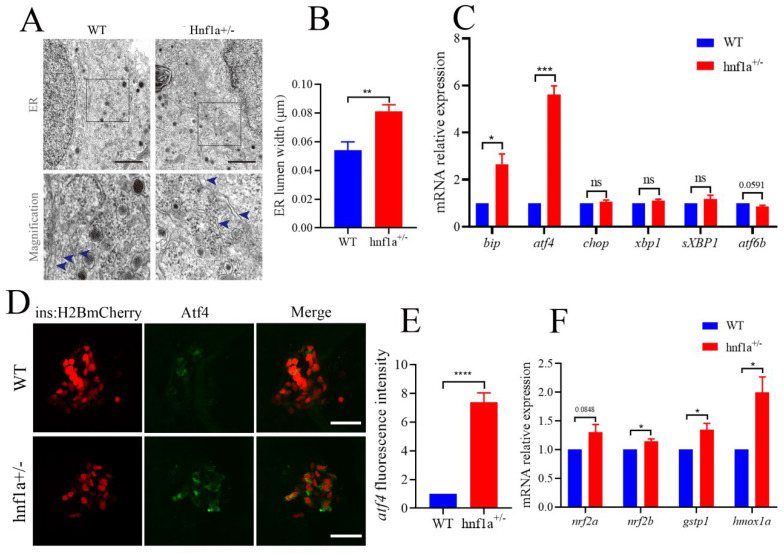
The β cells of *hnf1a^+/−^* zebrafish displayed ER stress. (**A**) Transmission electron microscopy analysis of ER in WT and *hnf1a^+/−^* β cells at 6 dpf zebrafish. Blue arrows indicated magnified ER morphology. Scale bar: 3 μm. (**B**) Quantification of ER lumen width for WT and *hnf1a^+/−^* β cells. At least 15 different locations of the same cell were measured; *n* = 3 β cells for each genotype. (**C**) RT-qPCR analysis of the mRNA expression levels for ER-stress-related marker genes in WT and *hnf1a^+/−^* larvae at 6 dpf: *bip, atf4, chop, xbp1, sXBP1*, and *atf6b*. (**D**) Immunofluorescence analysis of WT and *hnf1a^+/−^* larvae at 6 dpf with atf4 staining (Green). β cells are indicated by the red fluorescence with Tg(ins:H2BmCherry). Scale bar indicates 20 μm. (**E**). Quantification of fluorescence intensity for atf4 in WT and *hnf1a^+/−^*; *n* = 3 larvae for each genotype. (**F**) RT-qPCR analysis of the mRNA expression levels for *nrf2a, nrf2b, gstp1*, and *hmoxla* in WT and *hnf1a^+/−^* larvae; *n* = 3 larvae for each genotype. Results are represented as means with standard errors; * *p* < 0.05, ** *p* < 0.01, and *** *p* < 0.001, and **** *p* < 0.0001. Student’s *t*-test. All experiments were performed in at least three biological repeats. WT, wild type.

In the TEM images, we also carefully examined the ultrastructure of β cells. Interestingly, the ER lumen of *hnf1a^+/−^* zebrafish was dilated compared with the WT in β cells (Figure 3A,B). We then further analyzed the marker genes in the ER stress pathway. As shown in Figure 3C, *bip* and *atf4* were significantly upregulated, and *atf6b* was downregulated, while *xbp1, sXbp1* (spliced xbp1), and chop were not changed. This suggested that the *hnf1a^+/−^* might induce ER stress through activating Atf4 without inducing apoptosis. A further investigation showed that the Atf4 staining signal was stronger in *hnf1a^+/−^* zebrafish β cells compared to WT zebrafish (Figure 3D,E).

Additionally, chemically induced ER stress could activate Nrf2 in some mammalian cultured cells [25]. Furthermore, the antioxidant transcription factor nuclear factor erythroid 2 related factor 2 (Nrf2) was downstream of PERK, an unfolded protein response (UPR) signal pathway. PERK also phosphorylates and activates Nrf2 [26]. Hence, we detected the transcription levels of *nrf2a, nrf2b, gstp1*, and *hmox1a*. As shown in Figure 3F, *hnf1a^+/−^* decreased the transcription level of marker genes for Nrf2. These data suggested that ER stress was induced in the β cells of *hnf1a^+/−^*.

### 2.4. Overexpression of HNF1a-Q125ter Variant Led to β-Cell Dysfunction In Vitro

**Figure 4:**
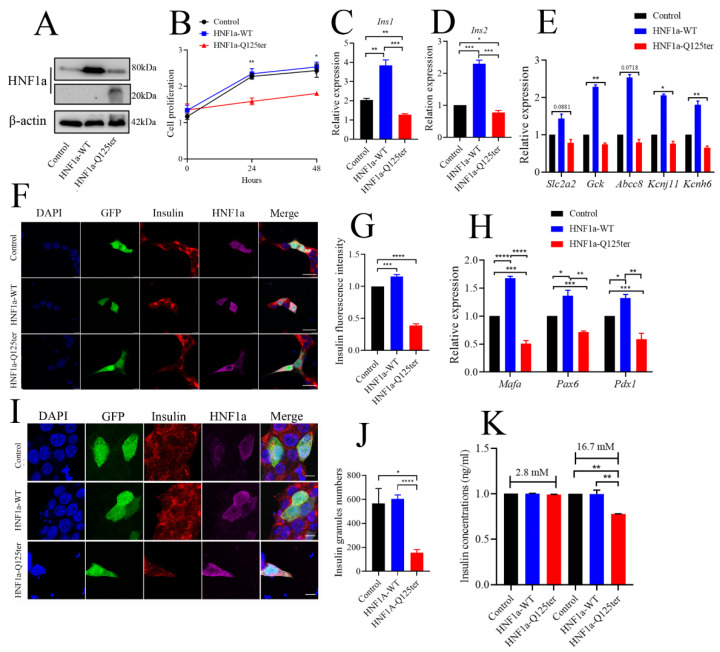
HNF1a-Q125ter impaired β-cell function in vitro. (**A**) Western blot analysis of HNF1a in INS-1 cells after transfection with control, HNF1a-OE, or HNF1a-Q125ter plasmids for 24 h. (**B**) The cell-proliferation analysis of control, HNF1a-WT, or HNF1a-Q125ter plasmid transfected Ins-1 cells at 0, 24, and 48 h. (**C**,**D**) RT-qPCR analysis of the expression levels of insulin genes *Ins1* (**C**) and *Ins2* (**D**) in Ins-1 cells after plasmid transfection for 24 h. (**E**) RT-qPCR quantification of insulin-secretion markers in control, HNF1a-WT, and HNF1a-Q125ter after plasmid transfection for 24 h: *Slc2a2, GCK, Abcc8, Kcnj11,* and *Kcnh6*. (**F**,**G**) Representative immunofluorescence images (**F**) and quantification of insulin fluorescence (**G**) of insulin content from control, HNF1a-WT, and HNF1a-Q125ter transfected Ins-1 cell. Insulin was stained with red, and HNF1a was stained with Magenta; GFP indicates the plasmid-transfected positive cells and DAPI-stained nuclei. Scale bar indicates 20 μm. (**H**) RT-qPCR quantification of β-cell maturation and differentiation markers (*Mafa, Pdx1,* and *Pax6*) in control, HNF1a-WT, and HNF1a-Q125ter after transfection for plasmids at 24 h. (**I**,**J**) Immunofluorescence analysis of insulin granules in different plasmid-transfected Ins-1 cells. The representative images (**I**) and quantification (**J**) of insulin granules are displayed. Insulin stained with red, HNF1a stained with magenta, GFP indicates the plasmid-transfected positive cells, and DAPI is stained nuclei. Scale bar indicates 40 μm. (**K**) Glucose-stimulated insulin secretion analysis in Ins-1 cells transfected with different plasmids. Results are represented as means with standard errors: * *p* < 0.05, ** *p* < 0.01, *** *p* < 0.001, and **** *p* < 0.0001. One-way ANOVA. All experiments were performed at least three times, unless otherwise indicated. WT, wild type.

To explore the detailed mechanism of HNF1a-Q125ter variant in β-cell function, we generated a HNF1a-Q125ter construct and expressed these constructs in the β-cell line, Ins-1 832/13, where overexpression of HNF1a-Q125ter suppressed Ins-1 cell growth, but not the HNF1a-WT (Figure 4A,B).

We also evaluated the effect of HNF1a-Q125ter overexpression on insulin synthesis in Ins-1 cells. As shown in Figure 4C,D, both *Ins1* and *Ins2* transcriptional levels were increased in HNF1a-WT-overexpressed cells, while they decreased in HNF1a-Q125ter-overexpressed cells. Moreover, the mRNA of several key transcription factors in the regulation of insulin biosynthesis were decreased (Figure 4H). Immunostaining also indicated that the insulin protein intensity was alleviated in HNF1a-WT-overexpressed cells, but reduced in HNF1a-Q125ter-overexpressed cells (Figure 4F,G). A similar trend was also observed in the proinsulin detected by immunoblot (Supplementary Figure S2A,B).

To elucidate the effect of HNF1a-Q125ter on insulin secretion, we first measured the insulin granules through immunofluorescent staining. Consistent with the result in *hnf1a^+/−^* zebrafish, HNF1a-Q125ter overexpression resulted in decreased insulin granules (Figure 4I,J). Glucose-stimulated insulin secretion assay was also applied to test the function of HNF1a-Q125ter. Ins-1 cells transfected with HNF1a-Q125ter showed blunted insulin secretion in response to high glucose (16.7 mM) (Figure 4K). Additionally, the transcription levels of markers for insulin secretion, e.g., *Slc2a2*, *Gck*, *Abcc8*, *Kcnj11*, and *Kcnh6*, were downregulated (Figure 4E). All of the above data suggested that overexpression of HNF1a-Q125ter led to β-cell dysfunction in Ins-1 cells, as was consistent with the phenomena in zebrafish. 

### 2.5. The HNF1a-Q125ter Variant Induced ER Stress by Activating the PERK/eIF1a/ATF4 Signaling Pathway

**Figure 5:**
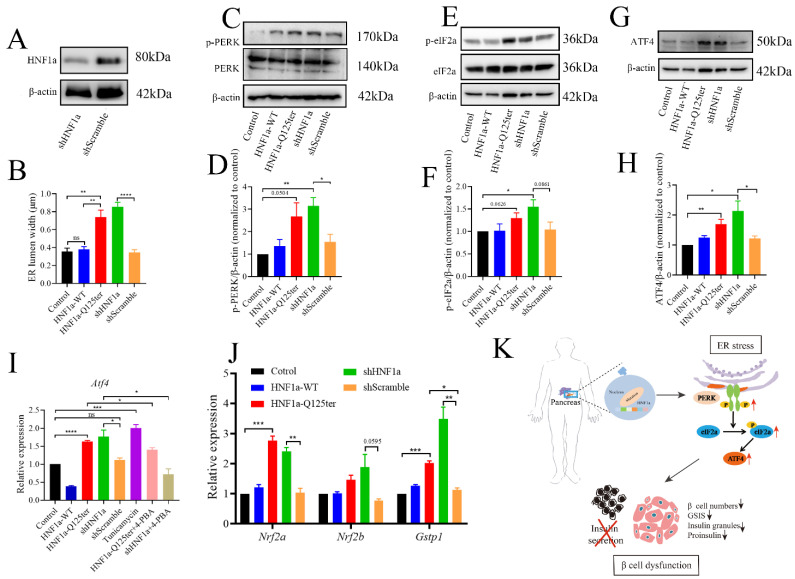
HNF1a-Q125ter induced ER stress through PERK/eIF2a/ATF4 signaling pathway. (**A**) Western blot of HNF1a in Ins-1 cells transfected with shHNF1a or control construct for 24 h. (**B**) Quantification of ER lumen width for control, HNF1a-WT, HNF1a-Q125ter, shScramble, and shHNF1a transfected Ins-1 cells, at least 15 different locations of the same cell were measured, *n* = 5 individual cells for each group. (**C**,**D**) Representative Western blot images (**C**) and quantification analysis (**D**) of p-PERK and PERK in different plasmids transfected Ins-1 cell. (**E**,**F**) Representative Western blot images (**E**) and quantification analysis (**F**) of p-eIF2a and eIF2a in different plasmids transfected Ins-1 cell. (**G**,**H**) Representative Western blot images (**G**) and quantification analysis (**H**) of ATF4 in different plasmid-transfected Ins-1 cell. Protein levels were normalized to β-actin. (**I**) RT-qPCR analysis of the expression levels of Atf4 mRNA level in different plasmids transfected Ins-1 cell. The tunicamycin treatment (5 μg/mL) was used as a positive control for ER stress, and chemical chaperone 4-PBA (300 μm/L) was used as an ER-stress reliever. (**J**) RT-qPCR analysis of the expression levels of Nrf2a, Nrf2b, and Gstp1 mRNA levels in different plasmids transfected Ins-1 cell. (**K**) Working model for the molecular mechanism of new HNF1a variant resulted in β-cell dysfunction. A schematic that showed how the new variant of HNF1a resulted in MODY3. HNF1a-Q125ter induced ER stress through upregulated p-PERK/p-eIF2a/ATF4 and resulted in β-cell dysfunctions, including decreased β-cells numbers, decreased insulin expression, reduced insulin granules, and impaired insulin secretion. Results are represented as means with standard errors; * *p* < 0.05, ** *p* < 0.01, *** *p* < 0.001, and **** *p* < 0.0001. One-way ANOVA. All experiments were conducted at least three times. WT, wild type; ER, endoplasmic reticulum; PERK, protein kinase R-like ER kinase; eIF2a, eukaryotic initiation factor 2; ATF4, activating transcription factor 4.

To gain a deeper understanding of HNF1a-Q125ter, we introduced the HNF1a knockdown (shHNF1a) plasmid to further explore the related mechanism. We confirmed that shHNF1a efficiently knocked down HNF1a in Ins-1 cells (Figure 5A). Since a similar HNF1a-Q125ter variant induced β-cell ER stress in zebrafish, we then questioned whether ER stress also occurred in Ins-1 cell. We imaged the ER morphology in Ins-1 cells transfected with HNF1a-WT, HNF1a-Q125ter, and shHNF1a by TEM (Supplementary Figure S3A). We measured the width of the ER lumen and found significantly dilated ER in cells overexpressed with HNF1a-Q125ter and shHNF1a, compared with the control or HNF1a-WT (Figure 5B and Supplementary Figure S3A). 

To investigate the mechanism for HNF1a-Q125ter- and shHNF1a-induced ER stress, we tested three UPR signaling pathways and found no effects for XBP1 and ATF6 pathways (Supplementary Figure S3B,C). Remarkably, we found the PERK/eIF2a/ATF4 pathway was activated, as protein levels of p-PERK, p-eIF2a, and ATF4 were increased in HNF1a-Q125ter- and shHNF1a-expressed Ins-1 cell that were not treated with tunicamycin and 4-PBA (Figure 5C–H). Moreover, Atf4 mRNA levels were upregulated in HNF1a-Q125ter- and shHNF1a-expressed Ins-1 cell. Interestingly, the chemical chaperone 4-PBA (300 μm/L), which is an ER-stress reliever, significantly suppressed the expression of Atf4 in HNF1a-Q125ter- and shHNF1a-expressed Ins-1 cell (Figure 5I). Additionally, the downstream signals of PERK, *Nrf2a, Nrf2b*, and *Gstp1*-were also decreased (Figure 5J). Taken together, these results suggest that HNF1a-Q125ter and shHNF1a work in a similar manner, which induced ER stress through the PERK/eIF1a/ATF4 signaling pathway.

## 3. Discussion

The HNF1a variant that causes MODY3 is the most commonly reported MODY, comprising 30% to 65% of all MODY cases. However, the molecular mechanisms that impair islet β-cells function are still unclear. In this study, we found a new HNF1a variant, HNF1a-Q125ter, in a human, presenting with atypical clinical symptoms of MODY3 are non-sensitive to sulfonylureas, and explored its molecular mechanisms by using zebrafish and the Ins-1 cell line. 

We firstly generated a similar variant in zebrafish (*hnf1a^+/−^*), and the animal displayed hyperglycemia, which was a diabetic phenomenon (Figure 2D). We further found that the *hnf1a^+/−^* significantly decreased the zebrafish β-cell numbers and that overexpression of HNF1a-Q125ter suppressed Ins-1 cell growth (Figure 2B and Figure 4B). Consistent with our data, several HNF1a variants, including p.D80V, p.R203C, p.P475L, and p.G554fsX556, resulted in the retardation of Ins-1 cell growth by inducing cell-cycle arrest at the transition from G1 to S phase [27]. Additionally, Ins-1 cells overexpressing the dominant-negative variant HNF1a-P291fsinsC showed significant growth impairment, which is due to delayed transition from the G1 to S phase and was mainly manifested by downregulation of cyclin E and upregulation of P27 [28]. Induction of another dominant-negative HNF1a variant (SM6) suppressed cell-cycle progression by increasing the level of the mTORC1-regulated cell-cycle inhibitors [29]. However, the patient with HNF1a^T260M^ showed impaired GSIS but no obvious decrease in β-cell mass, and neither β-cell proliferation nor apoptosis [9]. Although the data on β-cell mass were inconclusive for the HNF1a variant, this was likely due to differences in the HNF1a mutation sites or species. 

Our data further suggested that insulin expression and secretion were also affected in HNF1a-Q125ter-overexpressed Ins-1 cells or *hnf1a^+/−^* zebrafish (Figure 2 and Figure 4 and Supplementary Figures S3 and S4). Similarly, Hnf1a KO mice (*Hnf1a^−/−^*) displayed decreased insulin levels and impaired GSIS [12]. Moreover, the human HNF1a variant HNF1a^T260M^ also showed impaired islet GSIS, which disturbed the transcriptional regulatory network of insulin secretion [9]. Studies also revealed that HNF1a directly regulated transcription of several genes essential for insulin secretion, e.g., Slc2a2 and HNF4a [30,31]. In the clinic, MODY3 patients with the HNF1A variant had impaired insulin secretion, including reduced fasting insulin levels and decreased OGTT insulin levels [32]. Taken together, these data suggest a consensus that disruption of HNF1a results in impaired insulin secretion across species, from zebrafish to mice to humans. 

We further showed that the HNF1a-Q125ter variant’s induced β-cell dysfunction could be due to activation of ER stress. Several pieces of evidence support our observation, including upregulated ER stress markers (Atf4 and Nrf2 [33]), and dilation of ER lumen (Figure 3 and Figure 5). Congruent with our results, the target expression of dominant-negative HNF1a variant (SM6) in mouse β cells dilated rough ER cisternae [34]. In addition, overexpression of HNF1a variant (SM6) in Ins-1 cell lines resulted in ER stress mainly by downregulation of XBP1 and BiP [35]. In this study, we demonstrated that the HNF1a-Q125ter variant induced ER stress by activating the PERK/eIF1a/ATF4 signaling pathway (Figure 5). Although the downstream signals were different in HNF1a-Q125ter, and SM6 variants induced ER stress, the differences might be caused by the variants’ sites. 

The HNF1a-Q125ter variant lost most of its DNA-binding domain and lost its entire transactivation domain, and the phenotype of HNF1a-Q125ter overexpression was highly similar to the knockdown (shHNF1a) phenotype. Moreover, the in vivo and in vitro functions of HNF1a-Q125ter variants are similar to other dominant-negative HNF1a variants (P291fsinsC and SM6). Hence, we speculated that the HNF1a-Q125ter variant might play its role in β-cell dysfunction in a dominant-negative manner.

In summary, we identified a novel HNF1a variant, HNF1a-Q125ter, that induced β-cell dysfunction. Both through in vivo and in vitro approaches, our data revealed that the HNF1a-Q125ter variant decreased β-cell numbers, reduced β-cell growth, and impaired insulin synthesis and secretion. Further investigations demonstrated HNF1a-Q125ter induced ER stress by activating the PERK/eIF2a/ATF4 signaling pathway. Future studies are needed to evaluate how the HNF1a variant interacts with molecules in the ER stress pathways and possible therapeutic targets to reduce the stress on β-cells for alleviating insulin-secretion defects.

## 4. Materials and Methods

### 4.1. Patient

Subjects were recruited from routine clinical activities. A 15-year-old male adolescent was diagnosed with MODY3 by Sanger sequencing technology, using exome-sequencing approaches. DNA was extracted from the peripheral blood of the patient by column method. The exon coding regions of ABCC8, AKT2, BLK, CEL, EIF2AK3, GCK, GLIS3, GLUD1, HADH, HNF1A, HNF1B, HNF4A, INS, INSR, KCNJ11, KLF11, MAPK8IP1, NEUROD1, PAX4, PDX1, PLAG1, PTF1A, RFX6, SLC19A2, SLC2A2, UCP2, ZFP57, and other genes were directly sequenced and compared with the reference sequence to find possible gene variants. The database of common genetic variants in this study included HGMD (Human Gene Mutation Database), ClinVar, ESP6500, 1000 genomics, dbSNP, and UniPort. 

### 4.2. Zebrafish Maintenance

Zebrafish (Danio rerio) were raised in an aquaculture system (Haisheng, China) on a 14:10 h light–dark cycle at 28 °C. Embryos were obtained and raised based on published standard methods [36]. The following transgenic lines were used in this study: Tg(-1.2ins:H2BmCherry) [37], Tg(Ins:GcaMP6s;cryaa:mCherry) [38], Tg(gcga:GFP) [39], Tg(pdx-1:GFP) [40], and LiPan (lfabf:ds-Red; elaA:EGFP) [41]. All animal experiments were performed according to local guidelines and regulations of the Xiamen University Institutional Animal Care and Use Committee (Protocol XMULAC20160089, 10 March 2016).

### 4.3. Establishment of hnf1a Variant Zebrafish Using CRISPR/Cas9 Technique 

HNF1a gRNA synthesis was conducted according to the standard protocol, as in a previous publication [42]. The sgRNA target site (ACAACCTTCCCCAGAGAG) was designed by using the online tool CRISPRscan, and sgRNA was synthesized from a T7 kit (MAXIscript T7 Transcription Kit, Invitrogen, Carlsbad, CA, USA). Then sgRNA and Cas9 protein (NEB, Beijing, China) were co-injected into single-cell-stage embryos.

The mutant F0 generation was raised to adulthood, and the F1 generation zebrafish were obtained by crossing with AB zebrafish. Genomic DNA prepared from adult fin clips was genotyped by PCR followed by 1% agarose gels and polyacrylamide gels, using the following primers: Forward primer: ATGCTTCACAAGTACATAATACA. Reverse primer: TTGAGGTGCTGCGACAGAT. The single mutant zebrafish with a 2 bp gene deletion was obtained by sequencing. 

### 4.4. Cell Culture and Transfection 

Ins-1 832/13 cells were cultured in RPMI-1640 supplemented with 10% heat-inactivated fetal bovine serum (FBS), 1 mM sodium pyruvate, 10 mM HEPES, 50 μM β-mercaptoethanol, 100 U/mL penicillin, and 100 μg/mL streptomycin. Cells were maintained at 37 °C in a 5% CO_2_ incubator. The cells were trypsinized by using 0.25% trypsin–EDTA. All experiments were performed by using Ins-1 cells that were 70–80% confluent.

Ins-1 cells were transfected with pIRES2-eGFP empty vector, pIRES2-HNF1a-Q125ter-eGFP, pIRES2-HNF1a-eGFP, pGIPZ-shScramble-eGFP, and pGIPZ-shHNF1a-eGFP, using lipofectamine 3000 (Invitrogen, L3000015). Cells were harvested for various assays 24 h after transfection. For RT-qPCR experiments of Atf4, following transfection, Ins-1 cells were overexpressed for HNF1a-Q125ter and shHNF1a, then treated with 5 μg/mL tunicamycin (ER stress inducer) or 300 μmol/L 4-PBA (ER stress inhibitor) for 12 h.

### 4.5. Islets Isolation

We isolated islets by collagenase digestion from larvae, as described [43]. Wild-type (WT) and hnf1a mutant larvae were anesthetized and digested in 250 μL collagenase P solution (0.6 mg/mL, dissolved in HBSS, Roche, Basel, Switzerland) for 5 min, at 37 °C. The digestion was then stopped by adding 1 mL stop solution (10% FBS in HBSS). The lysate was spun, and the pellet was resuspended in cold HBSS plus 10% FCS. The suspension was transferred to a Petri dish, and the islets were picked under Leica M205 FCA fluorescence stereomicroscopy (Leica, Wetzlar, Germany).

### 4.6. RNA Extraction and Quantitative RT-qPCR 

Total RNA from Ins-1cells, islets, and larvae was extracted by using the RNA Simple Total RNA Kit (Tiangen, DP419, Beijing, China). Reverse transcription was performed by using the FastKing RT Kit (with gDNase) (Tiangen, KR116, Beijing, China). Then qPCR-RT was carried out by using 2× SYBR Green PCR Master Mix (Lifeint, Xiamen, China), and the 2^−ΔΔct^ method was used to calculate the gene-expression fold change normalized to Ct values of β-actin or 18s of the control sample. Primer sequences are listed in Supplementary Table S1.

### 4.7. Western Blot

Cells were washed in ice-cold PBS and subsequently homogenized in 1× RIPA buffer (Sigma, R0728) supplemented with protease inhibitors (MCE, HY-K0010) and phosphatase inhibitors (MCE, HY-K0021). The suspensions were centrifuged at 12,000 rpm, at 4 °C for 10 min. The supernatants were collected and assayed for total protein concentration by using the PierceTM BCA Protein assay kit (ThermoFisher Scientific, A23228, Massachusetts, USA). Samples were then electrophoresed on SDS–PAGE gel and transferred onto PVDF membranes. Membranes were incubated with the antibodies listed in Supplementary Table S2. Visual bands were detected by using ChemicDocTM Imaging System (BIO-RAD, 733BR2378). Densitometric analysis was performed by using Image J software (National Institutes of Health, Image J 1.8.0.345, win64).

### 4.8. β-Cell Counting and Imaging

Fluorescence-positive cell counting was conducted with reference to a previously published paper [44]. In brief, larvae were fixed in 4% paraformaldehyde overnight, at 4 °C, and then washed with PBST and flat mounted in Aqua-Mount (Richard-Allan Scientific, Massachusetts, USA), with their right sides facing the coverslip. The β-cell numbers were counted based on the nuclear mCherry signal numbers by a Zeiss Axiolmager A1 microscope (Carl Zeiss, Jena, Germany). All the counting was repeated by a blinded reviewer.

### 4.9. Immunofluorescence

HeLa cells and Ins-1 cells were seeded onto glass coverslips for transfection, as described above. Cells were fixed in 4% paraformaldehyde for 15 min and blocked in 5% FBS and 0.1% Tween-20 in 1× PBS for 2 h, at room temperature. Cells were stained with primary antibodies diluted in blocking solution overnight, at 4 °C, followed by secondary antibodies for 2 h, at room temperature. Finally, DAPI-Fluoromount-GTM (Yeasen Biotechnology, 36308ES11, Shanghai, China) was used to dye DAPI on glass slides.

The 6 dpf larvae were fixed in 4% paraformaldehyde overnight, at 4 °C. Larvae were washed in 1× PBS, dehydrated in methanol, frozen in methanol, redehydrated in methanol, frozen in acetone, and blocked in 5% FBS in PBDT for 2 h at room temperature. The larvae were stained with primary antibodies overnight, at 4 °C, followed by secondary antibodies for 2 h, at room temperature. Finally, larvae were laid on glass slides and compacted with glass coverslips. The Leica SP8 Confocal microscope was used for imaging. The brightness and contrast of entire images, where adjusted in some instances, were adjusted equally across all samples of the same experiment. The antibodies and further details are represented in Supplementary Table S3.

### 4.10. Glucose-Stimulated Insulin Secretion

Ins-1 cells were seeded at a density of 800,000 cells per well of a 12-well plate and transfected as described above. Cells were incubated in 2.8 mmol/L glucose in KRB buffer for 1 h, followed by 16.7 mmol/L glucose in KRB buffer for 1 h before analysis. The concentration of insulin in the supernatants was quantified by using the Rat/Mouse Insulin ELISA Kit (Millipore, EZRMI-13K).

### 4.11. Transmission Electron Microscopy

Ins-1 cells after intervention were placed in 2.5% glutaraldehyde for 30 min, at room temperature, followed by overnight at 4 °C. A total of 200 μL 20% BSA was added to cells, which were then collected. The suspensions were centrifuged at 2000 rcf, at room temperature, for 5 min, and mixed in phosphate buffer. WT and *hnf1a^+/−^* larvae were fixed in 2.5% glutaraldehyde overnight, at 4 °C, followed by isolating islets under Leica M205 FCA fluorescence stereomicroscopy. The islets were collected in 1% agarose gel. Images were analyzed under a Hitachi-HT-7800 transmission electron microscope. 

### 4.12. Live-Imaging of Calcium Influx

Zebrafish larvae were euthanized in cold Danio buffer for 3 min, after which the larvae were transferred to the extracellular solution (ECS) (containing 5 mM glucose), and the islets were isolated under the Leica M205 FCA fluorescence stereomicroscope with a syringe needle. We put a drop of melted 0.5% agarose on each of the glass-bottomed dishes beforehand; while waiting for the agarose to cool down to room temperature, we transferred the individual islets to dishes with a pipette and immersed them in 0.5% agarose. Adding ECS, we then carefully placed the dish on the plate holder of the Leica SP8 confocal microscope, using a 20× objective. Using the filter for red fluorescence to view the position of β-cell nuclei, we focused on the islet. The green channel recorded the GCaMP fluorescence intensity. After the first 50 frames (5 mM glucose), we increased the glucose concentration of the surrounding solution to 20 mM without stopping the recording. 

### 4.13. Statistical Analysis

Statistical analysis was performed by using GraphPad PRISM 8 software employing two-tailed Student *t*-tests to calculate *p*-values for unpaired comparisons between two groups, and one-way ANOVA was used for comparisons between three or more groups, using *p* < 0.05 to represent significance. All data were shown as mean ± SEM. The sample sizes of independent experiments can be found in the figure legends.

## Data Availability

The data that support the findings of this study are available within the article and its Supplementary Materials.

## Supplementary Materials

The following supporting information can be downloaded at https://www.mdpi.com/article/10.3390/ijms232113022/s1.
