# Supplementary material for: Novel Loss-of-Function Variant in HNF1a Induces β-Cell Dysfunction through Endoplasmic Reticulum Stress
Source: Int J Mol Sci. 2022 Oct 27;23(21):13022. doi: 10.3390/ijms232113022 (PMC9656704; doi:10.3390/ijms232113022)
Supplement: Supplementary file 1 [file ijms-23-13022-s001.zip › Sequencing comparison.pdf]

```
HumanWT           ----------MVSKLSQLQTELLAALLESGLSKEALIQALGEPGPYLLAGEGPLDKGESC
HumanMutant       ----------MVSKLSQLQTELLAALLESGLSKEALIQALGEPGPYLLAGEGPLDKGESC
ZebrafishWT       MDGGESRRSEGSGRLSALQEQLVWSLLGSGLSKELLIQAMG-----------DLERERAS
ZebrafishMutant   MDGGESRRSEGSGRLSALQEQLVWSLLGSGLSKELLIQAMG-----------DLERERAS
                              .:** ** :*: :** ****** ****:*            *:: .:.
HumanWT           GGGRGELAELPNGLGETRGSEDETDDDGEDFTPPILKELENLSPEEAAHQKAVVETLLQE
HumanMutant       GGGRGELAELPNGLGETRGSEDETDDDGEDFTPPILKELENLSPEEAAHQKAVVETLLQE
ZebrafishWT       SGGER----------ADRADCESSEEGEMDNPPPIYRELEKLPPEEARQRAQVDQLLQE
ZebrafishMutant   SGGER----------ADRADCESSEEGEMDNPPPIYRELEKLPPEEARQRAQVDQLLQE
                  .**.             *.. :.::.   * .*** :***:*.*****:*:* *: ****
HumanWT           DPWRVAKMVKSYLQQHNIPQREVVDTTGLNQSHLSQHLNKGTPMKTQKRAALYTWYVRKQ
HumanMutant       DPWRVAKMVKSYLQ*---------------------------------------------
ZebrafishWT       DPWHVAKMVKSYMQQHNLPQREVVESTGLNQSHLSQHLNKGTPMKNQKRAALYSWYTKKQ
ZebrafishMutant   DPWHVAKMVKSYMQQHNLPQR*--------------------------------------
                  ***:********:*
HumanWT           REVAQQFTHAGQGGLIEEPTGDELPTKKGRRNRFKWGPASQQILFQAYERQKNPSKEERE
HumanMutant       ------------------------------------------------------------
ZebrafishWT       AEISQQFTNASRGVMSGEEPGEDV--RKGRRNRFKWGPASLQILFQAYERQKNPSKEERE
ZebrafishMutant   ------------------------------------------------------------

HumanWT           TLVEECNRAECIQRGVSPSQAQGLGSNLVTEVRVYNWFANRRKEEAFRHKLAMDTYSGPP
HumanMutant       ------------------------------------------------------------
ZebrafishWT       GLVEECNRAECLQRGVSPSQLAGLGSNLVTEVRVYNWFANRRKEEAFRHKLALDVPYSSQ
ZebrafishMutant   ------------------------------------------------------------

HumanWT           PGPGPGPALPAHSSPGLPPPALSPSKVHGVRYGQPATSETAEVPSSSGGPLVTVSTPLHQ
HumanMutant       ------------------------------------------------------------
ZebrafishWT       SAASTCQTLPSSPSPGLKYSQTVVCESLGTVRSSGGEDRGASVRLASPVQLEPS------
ZebrafishMutant   ------------------------------------------------------------

HumanWT           VSPTGLEPSHSLLSTEAKLVSAAGGPLPPVSTLTALHSLEQTSPGLNQQPQNLIMASLPG
HumanMutant       ------------------------------------------------------------
ZebrafishWT       ---------HTLLETHHHKPASVGGSLPPVSTLTSLHGMSGSSA----------------
ZebrafishMutant   ------------------------------------------------------------

HumanWT           VMTIGPGEPASLGPTFTNTGASTLVIGLASTQAQSVPVINSMGSSLTTLQPVQFSQPLHP
HumanMutant       ------------------------------------------------------------
ZebrafishWT       ------GAPGLFIPSVMSLGDSSLLIGLTSSQPQTVPIINNVGGGFTTLQPISFQQPLHA
ZebrafishMutant   ------------------------------------------------------------

HumanWT           SYQQPLMPPVQSHVTQSPFMATMAQLQSPHALYSHKPEVAQYTHTGLLPQTMLITDTTNL
HumanMutant       ------------------------------------------------------------
ZebrafishWT       SPQQPIAQQLQSHIAPSSFMATMAQLP---CHMYSKADLSSYPSSSLLSQAMVIADSSSI
ZebrafishMutant   ------------------------------------------------------------
```

```
HumanWT           SALASLTPTKQVFTSDTEASSESGLHTPASQATTLHVPSQDPASIQHLQPAHRLSASPTV
HumanMutant       ------------------------------------------------------------
ZebrafishWT       GTLTNLTAVRQILTTDPEGHTESAIEEESLHLQSTSPEPGSSGSLDLYPQSQTSETHSSH
ZebrafishMutant   ------------------------------------------------------------

HumanWT           SSSSLVLYQSSDSSNGQSHLLPSNHSVIETFISTQMASSSQ
HumanMutant       -----------------------------------------
ZebrafishWT       LQLSSPAGDIDPYIPAQMVSTAQ------------------
ZebrafishMutant   -----------------------------------------
```

- Black ***** indicates the amino acid sequences of the four groups were consistent. Red ***** indicates that truncation begins at this amino acid site.
