# Supplementary material for: Novel Loss-of-Function Variant in HNF1a Induces β-Cell Dysfunction through Endoplasmic Reticulum Stress
Source: Int J Mol Sci. 2022 Oct 27;23(21):13022. doi: 10.3390/ijms232113022 (PMC9656704; doi:10.3390/ijms232113022)
Supplement: Supplementary file 1 [file ijms-23-13022-s001.zip › SM Figures.pdf]

Figure legends

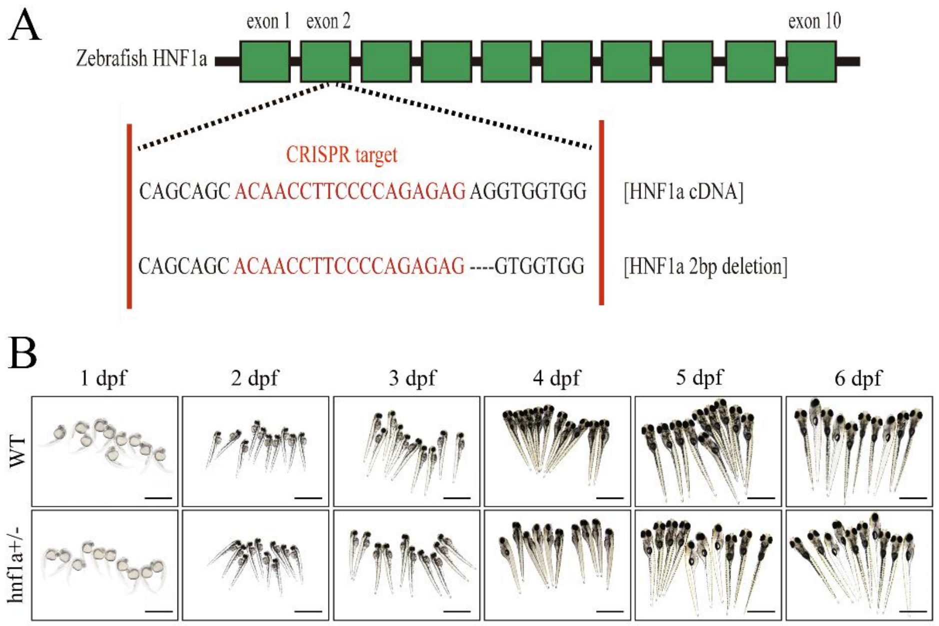

Figure S1. CRISPR-Cas9 target of *hnf1a*$^{+/-}$ and embryo morphology for WT and *hnf1a*$^{+/-}$. A. CRISPR-Cas9 target of *hnf1a*$^{+/-}$. **B**. Embryo morphology of WT and *hnf1a*$^{+/-}$ at 1dpf, 2 dpf, 3 dpf, 4 dpf, 5 dpf and 6 dpf. Scale bar: 20 μm. Each group contained 50 embryos. Error bars denote SEM. *$p < 0.05$, **$p < 0.01$, ***$p < 0.001$, ****$p < 0.0001$. Student's *t*-test. All experiments were performed at least three times unless otherwise indicated. WT, wild-type.

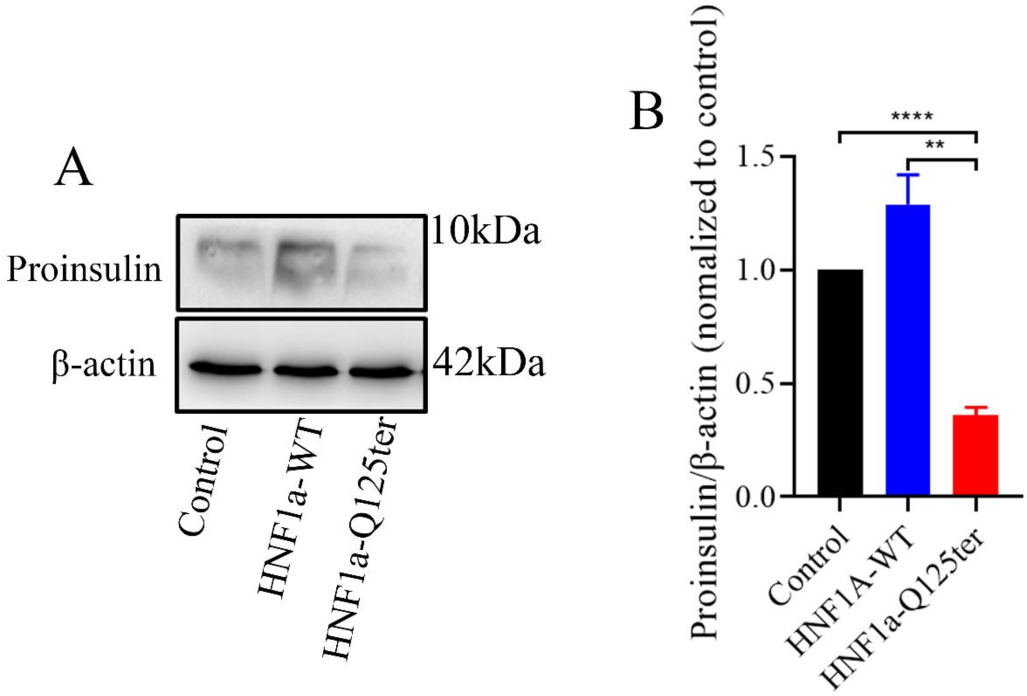

Figure S2. Protein level of Proinsulin. A-B. Western blot analysis (A) and quantification (B) of Proinsulin protein levels in Ins-1 cells after transfected with plasmid. n=3 larvae for each genotype. Error bars denote SEM. *$p < 0.05$, **$p < 0.01$, ***$p < 0.001$, ****$p < 0.0001$. Student's *t*-test. All experiments were performed at least three times unless otherwise indicated. WT, wild-type.

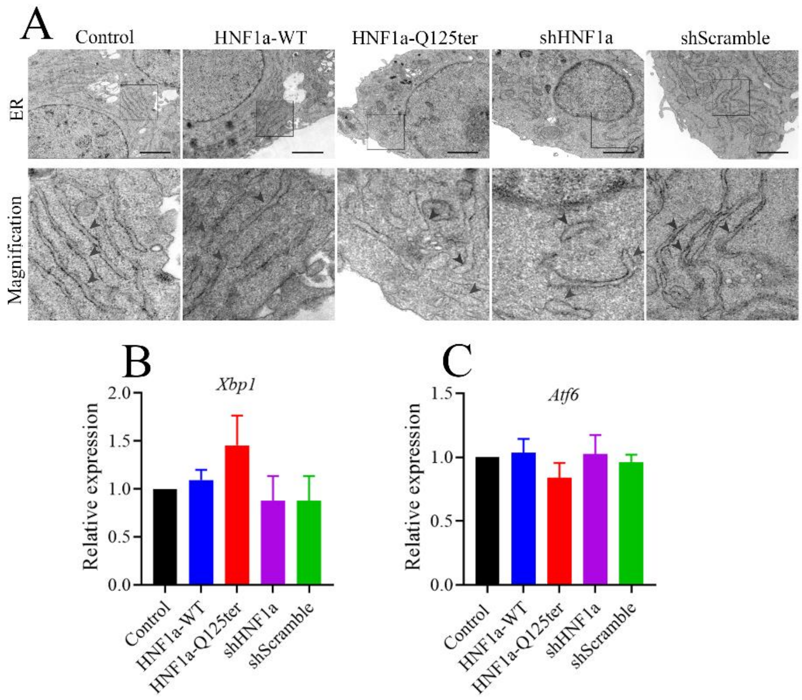

Figure S3. ER morphology and ER stress markers (*Xbp1* and *Atf6*). A. Transmission electron microscopy of ER morphology for control, HNF1a-WT, HNF1a-Q125ter, shScramble, and shHNF1a transfected plasmids after 24 hr. Black arrows indicated ER morphology. Scale bar: 2 µm. n=5 individual cells for each group. **B**. RT-qPCR quantification of *Xbp1* mRNA level in control, HNF1a-WT, HNF1a-Q125ter, shScramble, and shHNF1a transfected plasmids after 24 hr. **C**. RT-qPCR quantification of *Atf6* mRNA level in control, HNF1a-WT, HNF1a-Q125ter, shScramble, and shHNF1a transfected plasmids after 24 hr. Error bars denote SEM. One-way ANOVA. All experiments were conducted at least three times with Ins-1 cells unless otherwise indicated. WT, wild-type; ER, endoplasmic reticulum.

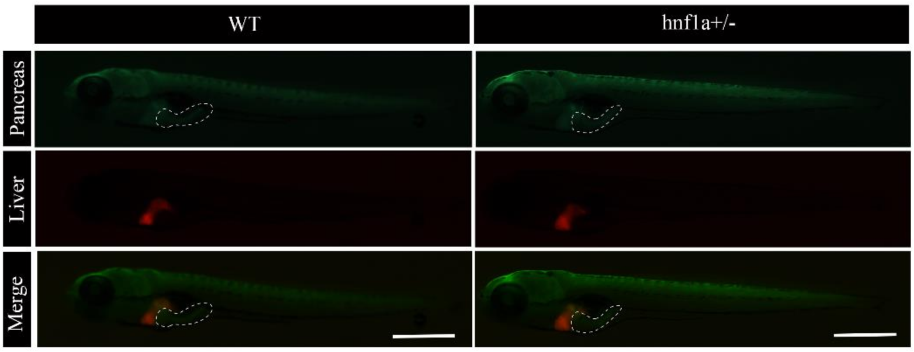

Figure S4. Exocrine pancreas in *hnf1a*$^{+/-}$ zebrafish. The zebrafish line was LiPan (lfabf:ds-Red; elaA:EGFP), which was used to assess the exocrine pancreas. The exocrine pancreas-specific GFP expression through a GFP filter (Green), and the liver-specific RFP expression through an RFP filter (Red). Exocrine pancreas morphology of WT and *hnf1a*$^{+/-}$ at 6 dpf. Scale bar: 50 µm.
