# Supplementary material for: Novel Loss-of-Function Variant in HNF1a Induces β-Cell Dysfunction through Endoplasmic Reticulum Stress
Source: Int J Mol Sci. 2022 Oct 27;23(21):13022. doi: 10.3390/ijms232113022 (PMC9656704; doi:10.3390/ijms232113022)
Supplement: Supplementary file 1 [file ijms-23-13022-s001.zip › SM Tables.pdf]

Table S1. RT-qPCR primers in vivo and in vitro

Gene	Forward primers (5’ to 3’)	Reverse primers (5’ to 3’)
**In vitro**		
18s	TCCGATAACGAACGAGAC	CTAAGGGCATCACAGACC
Ins1	CCCGTCGTGAAGTTGGAGGA	CAGTTGGTAGAGGGAGCAGAT
Ins2	TTTGTCAAGCAGCAGGTTTGTG	CCAGTGCCAAGGTCTGAAGG
Scl2a2	GTGCTGCTGGATAAATTCGCC	ATTGCAGACCCAGTTGCTGA
Gck	GTCACCGACTGCGACATT	GTCCTCACTGCGGCTTT
Abcc8	CTTCTTATGCCCAAACC	TCCTTCCTGCGTGTCT
Kcnj11	CAGAGGACCCTACAGAGC	GGAGTGGATGCTTGTGAC
Kcnh6	TTGGGTTACTGAAGACGG	TAGAGGGCGGTGACATAC
Pdx1	CGGTGCCAGAGTTCAG	CCAGTCTCGGTTCCATT
Mafa	CAAGGAGGAGGTCATCCGAC	TCTCCAGAATGTGCCGCTG
Pax6	ACCAGTGTCTACCAGCCAATC	TCATAACTCCGCCCATTCA
Atf4	GAATGGCTGGCTATGGA	CATCTGGCATGGTTTCC
Atf6	CGAGGGAGAGGTGTCTGTTTC	GTCTTCACCTGGTCCATGAGG
sXbp1	GAGTCCGCAGCAGGTG	CTCTGGGAGTTCCTCCAGACT
Gstp1	CCTGGGTCGCTCTTTA	TTGCCTCCCTGGTTCT
Nrf2a	TTGGGTGGTTTGGGTA	AGTAGTCGGCGTAGCAG
Nrf2b	ATCTGCTGTTCAGTTTGG	GCTTGGAATGGAGTGC
**In vivo**		
β actin	ACAGGGAAAAGATGACACAG	AGAGTCCATCACGATACCAG
insa	GTAAGCACTAACCCAGGCACA	GGGCAGATTTAGGAGGAAGG
insb	ATCCTGCTGCTGGCGTCTC	GCATCTGCTGCCTCATAACC
scl2a2	CTGGTCTTTATCCGTCG	CTCCCATAGCCCCTCT
gck	GATGCTTGTGAAAGTGGG	CCTTGAAGCCTTTAGTCC
abcc8	CTTCTTATGCCCAAACC	TCCTTCCTGCGTGTCT
kcnj11	TCTTCAGAACATAGTGGGTC	GGTGACGGTCTTTGTAC
kcnh6	AGATGAGCCTCAAACCA	CTTCATGCCTCCTATGC

pdx1	ATACCATCTCCCATTTCCG	TGCGCTCTGGTGTAAGCT
mafa	ATCAGCTCGTTACCATGTCCG	TCGCCCTCCAGTATGTGCC
pax6b	CCGAGATAGCGACGCCTGAA	CTCGGTTTAGGAGGAAGG
nkx6.1	TTGCGTGCTCACCACATCA	CTAGTCGGGCTCTTTCAGG
atf4	GTTGGAGGGCTCGTGC	GGGAACCGGGTGAATC
nrf2a	TCCACAGAAGCACCAACC	CGACCATATTTCCGCCAT
nrf2b	AGAGGGAGGAGGAGACCA	AGGGCAGACAACAAGGGA
homx1a	GCCCATTTACTTCCCTC	CCTGGGTCGCTCTTTA
atf6	CTGTGGTGAAACCTCCACCT	CATGGTGACCACAGGAGATG
atf6b	AGCCGCAAGAAGAAGAAGGAGT	CGAAGGTGAAGGTGATGAACAG
bip	AAGAGGCCGAAGAGAAGGAC	AGCAGCAGAGCCTCGAAATA
xbp1	GGGTTGGATACCTTGGAAA	AGGGCCAGGGCTGTGAGTA
sXbp1	TGTTGCGAGACAAGACGA	CCTGCACCTGCTGCGGACT
hnf1a	CGCAGCACCTCAACAA	CAGTCCCTCCCTTTCC

Table S2. Antibodies of western blot

**Antibodies**	**Brand**	**No.**
β-actin	Cell Signaling Technology	3700
Insulin	Cell Signaling Technology	8138
HNF1a	Cell Signaling Technology	89670
PERK	Cell Signaling Technology	5683
p-PERK	Cell Signaling Technology	3179
eIF2a	Abcam	ab5369
p-eIF2a	Abcam	ab32157
ATF4	Abcam	ab23760
	Proteintech	10835-1-AP
Goat anti-Rabbit IgG (H+L) Secondary Antibody	ThermoFisher Scientific	31460
Goat anti-Mouse IgM Secondary Antibody	ThermoFisher Scientific	31440

Table S3. Antibodies of immunofluorescence staining

**Antibodies**	**Brand**	**No.**
Insulin	Dako	A0564
HNF1a	Cell Signaling Technology	89670
ATF4	Proteintech	10835-1-AP
Alexa Fluor^{TM} 647 goat anti-rabbit IgG (H+L)	ThermoFisher Scientific	A21244
Alexa Fluor^{TM} 568 goat anti-guinea pig IgG (H+L)	ThermoFisher Scientific	A11075
Alexa Fluor^{TM} 488 goat anti-rabbit IgG (H+L)	ThermoFisher Scientific	A11008
