# Supplementary figures and images for: Novel Loss-of-Function Variant in HNF1a Induces β-Cell Dysfunction through Endoplasmic Reticulum Stress
Source: Int J Mol Sci. 2022 Oct 27;23(21):13022. doi: 10.3390/ijms232113022 (PMC9656704; doi:10.3390/ijms232113022)

## Slide 1
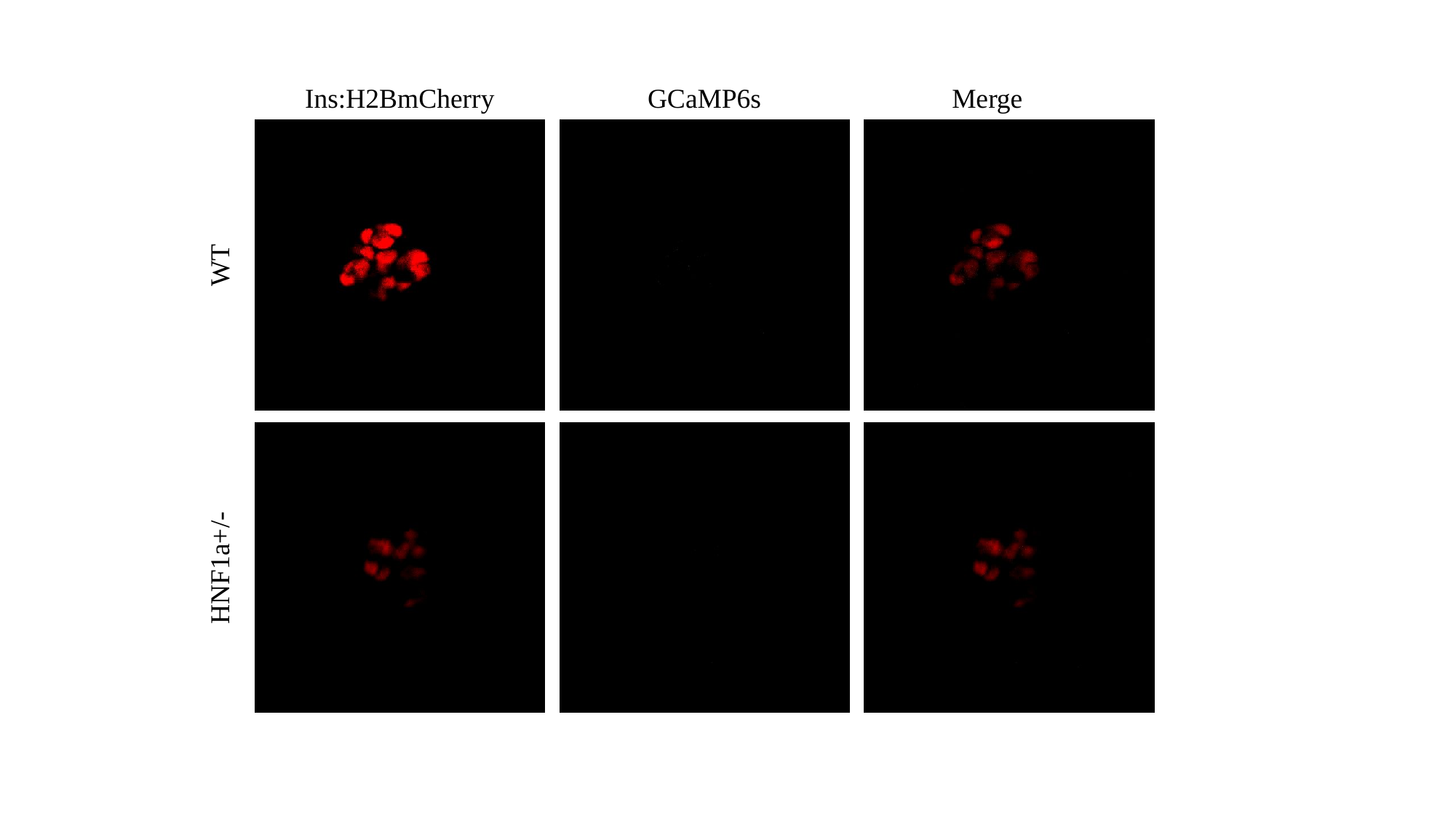

Ins:H2BmCherry
GCaMP6s
Merge
WT
HNF1a+/-

Supplement: Supplementary file 1 [file ijms-23-13022-s001.zip › SM Video for GCaMP6s.pptx]
